# Scleroderma-like Lesions in a Patient Undergoing Combined Pembrolizumab and Routine Chemotherapy: A Case Report and Literature Review

**DOI:** 10.3390/medicina60071092

**Published:** 2024-07-03

**Authors:** Hung-Liang Pai, Chin-Yin Liu, Ming-Hsin Yeh

**Affiliations:** 1 Department of Medicine, Chung Shan Medical University, Taichung City 40201, Taiwan; s0801003@gm.csmu.edu.tw; 2 Department of Dermatology, Chung Shan Medical University Hospital, Taichung City 40201, Taiwan; chinyin1983@gmail.com; 3 Division of Breast Surgery, Department of Surgery, Chung Shan Medical University Hospital, Taichung City 40201, Taiwan; 4 Institute of Medicine, Chung Shan Medical University, Taichung City 40201, Taiwan

**Keywords:** scleroderma-like lesion, triple-negative breast cancer, immune checkpoint inhibitors, taxane

## Abstract

Triple-negative breast cancer (TNBC) represents a challenging malignancy with limited treatment options and a poor prognosis. Adjuvant therapies, including chemotherapy and immune checkpoint inhibitors (ICI), are commonly employed following breast conservation surgery. However, these treatments can lead to various adverse effects, including cutaneous complications and connective tissue disorders. Here, we present the case of a 54-year-old woman with TNBC who developed morphea, a form of localized scleroderma, following adjuvant chemotherapy and pembrolizumab administration. This case highlights the rarity of drug-induced morphea and emphasizes the importance of recognizing and managing such adverse events in breast cancer patients. We discuss the clinical characteristics, diagnostic challenges, and treatment considerations associated with drug-induced scleroderma-like lesions, as well as the potential mechanisms underlying their development. Furthermore, we review the literature on the incidence, clinical features, and outcomes of scleroderma-like lesions induced by chemotherapy and ICIs. This case underscores the need for increased awareness of immune-related adverse events in patients receiving immunotherapy, as well as the importance of individualized treatment approaches to optimize patient care and outcomes.

## 1. Introduction

Triple-negative breast cancer (TNBC) is the most malignant type for women with the worst prognosis. Adjuvant therapies, including radiotherapy (RT), chemotherapy, and immune checkpoint inhibitors (ICI), are common treatments for TNBC patients who undergo breast conservation surgery. These clinical strategies have been investigated to be concurrent with several acute skin reactions and connective vascular diseases. Statistically, up to 90% of breast cancer patients who received RT suffered from skin complications [1]. These adverse effects, including erythema and confluent moist desquamation, particularly occur in tissues with rapidly dividing cells [2]. Chemotherapies have been identified as a contributing factor to scleroderma. For example, taxanes, recognized for their modulation of microtubule dynamics, are implicated in apoptosis induction and cytokine production enhancement. These pharmacological effects may contribute to tissue fibrosis similar to systemic sclerosis (SSc) [3]. ICI used in advanced triple-negative breast cancer are recognized contributors to secondary immune-related cutaneous adverse events (ircAEs), with reported incidence rates of up to 34% in patients treated with PD-1 inhibitors and between 43% to 45% in those receiving CTLA-4 inhibitors [4,5].

Scleroderma represents a rare entity within the spectrum of connective tissue disorders, characterized by significant cutaneous sclerosis and varying degrees of systemic involvement. SSc and localized scleroderma, also known as morphea, are the primary subtypes of scleroderma. SSc involves cutaneous sclerosis with visceral organ involvement, whereas morphea generally follows a benign course, with effects primarily confined to the skin [6]. Epidemiologic evidence indicates that the incidence of morphea is rare and predominantly occurs in women [7,8]. While the prevalence of morphea is similar across different age groups, there can be transitional forms from localized scleroderma to systemic sclerosis (SSc), particularly in adults [9]. Systemic sclerosis can be further categorized into two types: diffuse cutaneous systemic sclerosis (dcSSc) and limited cutaneous systemic sclerosis (lcSSc) [10]. Localized scleroderma includes several subtypes, such as linear scleroderma, plaque morphea, deep morphea, bullous morphea, and generalized morphea [11].

The association between breast cancer and scleroderma remains debated in the literature. An Italian study reported a significantly elevated standardized incidence ratio of 2.1 for breast cancer among scleroderma patients compared to the general population [12]. However, larger studies and meta-analyses have not consistently supported this finding, likely due to variations in study methodologies, demographic characteristics of studied populations, and regional factors [13,14,15,16].

Due to the rarity of drug-induced scleroderma-like lesions, comprehensive studies and case reports are scarce. Consequently, a thorough understanding of their incidence rate, clinical characteristics, treatment response, and long-term prognosis remains elusive [17]. This knowledge gap may cause diagnostic delays, which could hinder timely identification and therapeutic intervention, consequently predisposing patients to adverse outcomes such as breast disfigurement and chronic pain onset. In this case report, we describe a patient receiving adjuvant chemotherapy and pembrolizumab for breast cancer and subsequently developed morphea. We also comprehensively discuss the possibility of developing scleroderma-like lesions based on the drug administrations of the patient, along with the literature.

## 2. Case Presentation

A 54-year-old woman sought medical attention in 2023 after discovering a palpable mass in her right breast. During clinical examination, the mass was found to be firm and movable, devoid of tenderness, and accompanied by the absence of other breast abnormalities such as nipple discharge or deformity. The patient denied experiencing systemic symptoms commonly associated with breast malignancies, including fever, chills, weight loss, shortness of breath, chest pain, jaundice, blurred vision, or abdominal pain. Following the discovery of the breast mass, breast-conserving surgery was conducted with sentinel lymph node biopsy. Pathological analysis of the excised tissue confirmed a diagnosis of right breast cancer, characterized by estrogen receptor (ER) positivity in 5%, progesterone receptor (PR) negativity, and a Ki67 proliferation index of 45%. Additionally, the tumor displayed human epidermal growth factor receptor 2 (HER-2) negativity (score of 0+) and was classified as pT2N0M0, stage II. Notably, the margin involvement at the 3–6 o’clock position was less than 1 mm, indicating the need for further adjuvant treatment.

**Figure 1:**
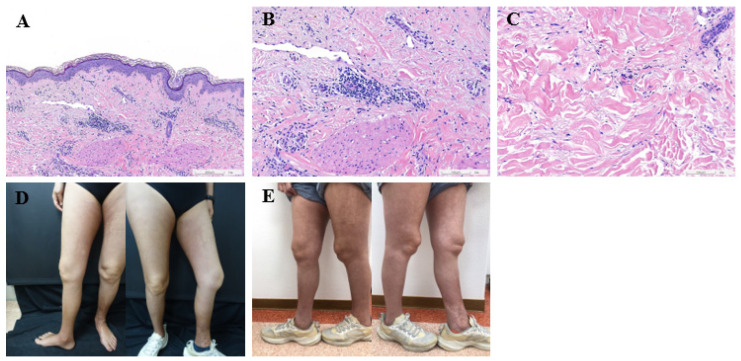
The representative images of lower extremities edema and drug-induced morphea. (**A**,**B**) The histological images of the patient’s skin lesions with perivascular lymphocytic infiltration. (**C**) The histological images of the patient’s skin lesions with thickened collagen bundles. (**D**) The representative images preceding the cessation of docetaxel administration. (**E**) The representative images after 6 months of discontinuation of docetaxel therapy.

**Figure 2:**
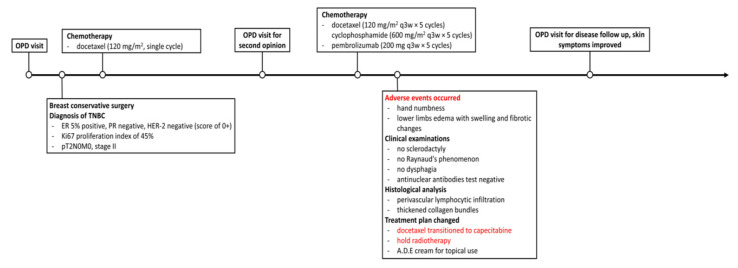
The graphical representation of the patient treatment courses.

Subsequent to the surgical intervention, she received adjuvant chemotherapy with a single cycle of docetaxel after the surgery in another institution. Seeking a second opinion at our outpatient department, she was advised to proceed with immunotherapy combined with chemotherapy and, thereafter, transition to docetaxel (120 mg/m^2^ q3w × 5 cycles) + cyclophosphamide (600 mg/m^2^ q3w × 5 cycles) + pembrolizumab (200 mg q3w × 5 cycles). Throughout the treatment course, the patient’s condition remained stable. However, after the completion of five cycles of combination treatment, the patient developed symptoms, including numbness in her hands and edema in her lower limbs. Additionally, skin symptoms such as swelling and fibrotic changes were observed in the bilateral lower extremities. Notably, clinical examination revealed the absence of sclerodactyly, Raynaud’s phenomenon, and dysphagia. Laboratory tests for antinuclear antibodies (ANA) were negative. Taken together, these findings led to the diagnosis of morphea rather than systemic sclerosis. The dermatological assessment also identified sclerotic changes in both lower limbs, with histological analysis showing perivascular lymphocytic infiltration and thickened collagen bundles (Figure 1). Although prescribed medication for symptomatic relief, the patient declined the use of methotrexate (MTX) and opted solely for the topical application of Sinpharderm A.D.E. Cream, a topical formulation containing vitamins A, D, and E. In light of the adverse events observed on the patient’s cutaneous tissue, the proposed course of radiotherapy was suspended. Instead, docetaxel was substituted with capecitabine. Six months subsequent to the cessation of docetaxel administration, a notable improvement in the morphea condition was observed (Figure 2).

## 3. Discussion

Drug-induced scleroderma-like lesions present with distinct clinical features that differentiate them from idiopathic SSc. These lesions commonly manifest initially in the lower extremities, particularly the lower legs, with sclerosis rarely extending to the trunk [18]. The progression typically involves an initial phase of edema followed by edematous sclerosis, which often initiates deep within the dermis, resembling conditions such as panniculitis or eosinophilic fasciitis. In cases where the fingers are affected, contractures may develop and significantly impact daily activities [17]. Taxane drugs, such as paclitaxel and docetaxel, have been associated with scleroderma-like lesions that exhibit unique pathophysiological pathways, including elevated serum interleukin-6 levels, with docetaxel exhibiting more pronounced effects [19]. Additionally, there’s a notable decrease in Friend leukemia integration 1 proteins in dermal fibroblasts, suggesting a differential fibrotic process compared to SSc [20]. Understanding these distinct pathways sheds light on the mechanisms underlying drug-induced scleroderma-like lesions and aids in clinical management. The accurate differentiation between drug-induced scleroderma-like lesions and SSc holds considerable clinical significance. Typically, scleroderma-like lesions are characterized by several distinct features, which include a temporal association between the administration of taxane-based agents and the onset of cutaneous sclerotic lesions [17]. These lesions are characterized by the absence of vascular complications like Raynaud’s phenomenon and lack of internal organ involvement, along with typically negative ANA. In contrast, SSc often exhibits Raynaud’s phenomenon of gastroesophageal reflux, indications of interstitial pneumonia, and concurrent positive ANA [21]. Generally, taxane-induced scleroderma-like lesions progress through three stages, including edema, a combination of edema and sclerosis, and sclerosis [22]. While precise diagnostic criteria for drug-induced scleroderma-like lesions remain undefined, a thorough evaluation of these clinical manifestations is imperative.

There are several treatment options for scleroderma-liked lesions. Topical therapies, including high-potency corticosteroids, tacrolimus, and imiquimod, effectively manage plaque morphea by reducing inflammation and symptoms [6]. Combination treatments like calcipotriol (a vitamin D analog) with corticosteroids further enhance outcomes, offering targeted strategies to mitigate disease progression and improve clinical manifestations in localized scleroderma [23]. Systemic treatments are essential when topical therapies are inadequate for morphea management. MTX, administered subcutaneously with folic acid supplementation, effectively improves skin lesions and halts disease progression [24]. It is often used alone or in conjunction with corticosteroids, balancing efficacy against manageable side effects. Mycophenolate mofetil (MMF) is an alternative for MTX-intolerant or inadequately responsive patients, although evidence from controlled studies is limited [25]. Other systemic options such as D-penicillamine, phenytoin, cyclosporine, and interferon are noted anecdotally or in small studies, with varied efficacy and potential adverse effects constraining their broader clinical use [26]. Phototherapy, including UVA with psoralens or narrowband UVB, is used to treat generalized and deep subtypes of morphea by modulating immune responses and collagen metabolism, potentially improving clinical outcomes. However, its efficacy and safety in morphea are based primarily on limited case reports and small studies, necessitating further research [27,28].

The advent of ICIs has revolutionized the landscape of oncological treatment by harnessing immune-mediated responses against tumor growth, particularly in metastatic melanoma, non-small-cell lung cancer, urothelial carcinoma, and TNBC [29]. However, the widespread adoption of ICIs, including nivolumab and pembrolizumab, has been associated with the induction of autoimmune conditions, including scleroderma-like lesions [30,31]. ircAEs associated with pembrolizumab and nivolumab encompass a diverse spectrum of dermatoses, ranging from relatively benign conditions such as vitiligo and morbilliform rash to severe entities like Stevens–Johnson syndrome and toxic epidermal necrolysis [32]. Notably, scleroderma has emerged as a less frequently reported but clinically significant adverse effect of ICIs, potentially complicating patient management and necessitating vigilance in monitoring. These sclerotic lesions tended to develop or worsen more rapidly with pembrolizumab compared to nivolumab, highlighting potential differences in the onset and severity of adverse events between these agents [33]. Additionally, pembrolizumab-induced morphea in breast cancer is exceptionally rare, with the majority of cases reported in melanoma and lung cancer patients receiving nivolumab [34]. Besides the PD-L1 treatments, ipilimumab combined with pembrolizumab and atezolizumab were also reported to cause morphea-like skin changes [35,36] (Table 1). Therefore, systemic immunosuppression must be balanced to reduce the risk of sclerotic lesion progression.

The combination of RT and ICI in TNBC shows promise, supported by the significant improvements in event-free survival demonstrated in the phase 3 KEYNOTE-522 study with pembrolizumab [45]. Concerns about toxicity have been raised, but recent evidence suggests comparable toxicity profiles between RT alone and RT with pembrolizumab, with challenges in managing specific adverse events such as myocarditis and metastatic non-small-cell lung cancer [46,47,48]. Minimizing cardiac exposure during adjuvant RT in patients with immuno-induced myocarditis is critical, emphasizing personalized treatment strategies and ongoing research into long-term risks [48]. While generally well tolerated, particularly in the adjuvant setting, the combination with PARP inhibitors can induce severe toxicity, such as in the treatment field of fibrosis [49]. Further investigation into patient-specific factors to optimize treatment outcomes and minimize toxicity is essential before widespread adoption of concurrent pembrolizumab and adjuvant RT [50]. 

The concurrent use of taxanes and RT has emerged as a significant strategy in the management of various cancers, including breast, lung, esophageal, and head and neck cancers. Both paclitaxel and docetaxel have shown efficacy in combination with RT, particularly in locally advanced disease settings, with modified paclitaxel dosing schedules demonstrating effectiveness in reducing skin reactions while maintaining treatment efficacy in neoadjuvant settings [51]. Mechanistically, paclitaxel enhances radiation sensitivity by promoting microtubule polymerization and inducing cell cycle arrest at the G2/M phase [52]. While the feasibility and tolerability of concurrent taxane and RT administration appear favorable, optimal scheduling remains uncertain, influencing treatment outcomes based on the timing of drug delivery relative to radiation exposure [53]. Differential toxicity profiles between paclitaxel and docetaxel should be considered in treatment decisions, with paclitaxel generally associated with lower overall toxicity, although management of potential adverse effects like treatment breaks remains critical, particularly in patients with advanced disease or post-mastectomy status.

In conclusion, our presented case underscores the significance of vigilance for ircAEs in patients following pembrolizumab administration with routine chemotherapy. The development of scleroderma-like lesions accentuates the necessity for clinicians to consider various etiologies, including ICI-related morphea and taxane-associated scleroderma, within the differential diagnosis framework. However, a significant limitation is the multitude of factors that may have contributed to the development of morphea-like skin changes, including docetaxel, pembrolizumab, and radiotherapy. Consequently, it is possible that these changes may have occurred coincidentally. Nonetheless, heightened awareness of such potential complications is imperative for informed decision-making regarding treatment strategies, emphasizing the pivotal role of tailored therapeutic approaches in optimizing patient care and outcomes.

## Figures and Tables

**Table 1:** Summary of different types of immune checkpoint inhibitor-induced scleroderma-like lesions.

Immune Checkpoint Inhibitor	Skin Lesions	Reference
Pembrolizumab	dcSSc	[34,37]
	lcSSc	[38]
	generalized morphea	[39,40]
Nivolumab	dcSSc	[41]
	lcSSc	[42]
	localized morphea	[43,44]
Pembrolizumab + ipilimumab	generalized morphea	[35]
Atezolizumab	SSc	[36]

dcSSc, diffuse cutaneous systemic sclerosis; lcSSc, limited cutaneous systemic sclerosis; SSc, systemic sclerosis.

## Data Availability

All data supporting the findings presented in this study are included in the article. Further details can be available upon request by contacting the corresponding author.
